# Management of breastefeeding for late preterm infants

**DOI:** 10.1186/1824-7288-40-S2-A36

**Published:** 2014-10-09

**Authors:** Elisa Civardi, Francesca Garofoli, Margherita Pozzi, Mauro Stronati

**Affiliations:** 1 Neonatal Intensive Care Unit, Fondazione IRCCS Policlinico San Matteo, Pavia, Italy; 2 Neonatal Immunology Laboratory, Fondazione IRCCS Policlinico San Matteo, Pavia, Italy

## 

Breast milk is the preferred feeding for all infants. Unfortunately, late preterm infants (LPIs) have a lower rate of feeding at-breast and lower expressed breast milk intake than other infants. [1] In fact, literature documents an increased risk of morbidity and even mortality of LPIs, often related to feeding problems, possibly due to an inadequate support of the breastfeeding. [2]

Born with low energy stores and high energy demands, LPIs may be sleepier and have more difficulty with latch, suck, and swallow. They are at risk for hypothermia, hypoglycemia, excessive weight loss, dehydration, failure to thrive, kernicterus, and breastfeeding failure.

Establishing breastfeeding in LPIs is problematic, due to neonatal physiological, neurological immaturity and due to maternal risk factors leading to delayed lactogenesis II. Mothers may be obese, experienced a cesarean delivery, have pregnancy induced hypertension, diabetes, or been treated for preterm labor. They easily experience anxiety about milk insufficiency and about separation from their babies for medical problems.

Sanitary staff has to encourage the immediate and extended skin-to-skin contact to improve postpartum stabilization of heart rate, respiratory effort, temperature control, metabolic stability, and early breastfeeding, possibly within 1 hour after birth. If the infant is healthy, it’s important to allow rooming in and free access to the breast.

It may be necessary to wake the baby up if he/she does not indicate hunger cues, which is not unusual in LPIs. The infant should be breastfed (even with expressed milk) 8 to 12 times/day. It is important observing the baby feeding at breast and showing the mother techniques to facilitate effective latch and adequate support of the neonate’s head. A nipple shield could be recommended. Pre- post-feeding weight may be helpful to assess milk transfer, because a supplementation with small quantities of maternal expressed milk, donor human milk, or formula may be necessary. If supplementing, the mother should pump milk after breastfeeding, 6 to 8 times/day to establish and maintain milk supply. [3]

LPIs developing complications are often discharged early, after successful transition to extra-uterine environment, but before lactogenesis II is fully established. Before discharge, adequate milk intake should be documented by feeding volume or by thriving. One/2 days after discharge, a follow up to check weight, feeding ability and jaundice is recommended.

In conclusion we may say that breastfeeding LPIs is possible, but to achieve this target, an adequate maternal support and a regular neonatal monitoring is required. [4]

## Competing interests

The authors declare that they have no competing interests.
